# Geologic controls on supercritical geothermal resources above magmatic intrusions

**DOI:** 10.1038/ncomms8837

**Published:** 2015-07-27

**Authors:** Samuel Scott, Thomas Driesner, Philipp Weis

**Affiliations:** 1 Department of Earth Sciences, Institute of Geochemistry and Petrology, ETH Zurich, Clausiusstrasse 25, 8092 Zurich, Switzerland

## Abstract

A new and economically attractive type of geothermal resource was recently discovered in the Krafla volcanic system, Iceland, consisting of supercritical water at 450 °C immediately above a 2-km deep magma body. Although utilizing such supercritical resources could multiply power production from geothermal wells, the abundance, location and size of similar resources are undefined. Here we present the first numerical simulations of supercritical geothermal resource formation, showing that they are an integral part of magma-driven geothermal systems. Potentially exploitable resources form in rocks with a brittle–ductile transition temperature higher than 450 °C, such as basalt. Water temperatures and enthalpies can exceed 400 °C and 3 MJ kg^−1^, depending on host rock permeability. Conventional high-enthalpy resources result from mixing of ascending supercritical and cooler surrounding water. Our models reproduce the measured thermal conditions of the resource discovered at Krafla. Similar resources may be widespread below conventional high-enthalpy geothermal systems.

## Abstract (web summary)

Utilizing supercritical geothermal water could multiply energy production, but the abundance, location and size of such resources is unclear. Here, the authors present numerical simulations and suggest that supercritical water may play a key role in removing heat from all magmatic intrusions.

## Graphical abstract

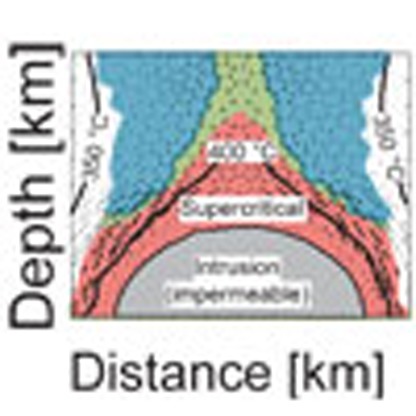

Heat loss from Earth's interior is elevated in magmatically active areas, where it is expressed at the surface through volcanic eruptions and geothermal geysers. Magmatic intrusions in the upper crust drive convection of meteoric groundwater^1^,^2^,^3^,^4^,^5^,^6^, resulting in boiling, high-enthalpy geothermal systems that can be conventionally exploited at an average of 3–5 MW electric power per well^7^. Heat transfer from a magmatic intrusion to groundwater occurs at depths below drilled geothermal reservoirs and, due to a lack of direct observation, the deep, very high-temperature parts of geothermal systems are not well understood.

Temperatures in the immediate vicinity of magmatic intrusions exceed the critical temperature of water, implying the possible occurrence of geothermal water as a single-phase, supercritical fluid. Thermodynamic considerations indicate that supercritical geothermal resources may be very favourable for power production^8^ and their theoretical response to production has been studied by numerical modelling^9^. In 2009–2012, an exploratory well drilled by the Iceland Deep Drilling Project (IDDP) penetrated a magma body at 2.1 km depth in the Krafla volcanic system and tapped an overlying reservoir of supercritical water at a temperature of 450 °C and enthalpy of 3.2 MJ kg^−1^, capable of generating 35 MW electricity from a single well^10^,^11^,^12^,^13^,^14^. In spite of studies of the magma^15^, well testing^14^ and modelling^13^, the thermo-hydraulic nature of the reservoir at Krafla has remained enigmatic.

The occurrence of comparable supercritical reservoirs in other magma-driven geothermal systems is hitherto unclear and exploration has been limited by a lack of understanding of the primary geologic factors that control the occurrence, depth, size and thermo-hydraulic properties of target reservoirs. Previous studies have shown that water enthalpy in conventional high-enthalpy geothermal systems depends strongly on the rock permeability^4^,^5^,^6^,^16^. The permeability of volcanic rocks and crystalline basement hosting geothermal systems^5^,^17^ is in the range of 10^−14^ to 10^−15^ m^2^, values hereafter referred to as ‘high' and ‘intermediate' permeability, respectively. High host rock permeability allows rapid fluid advection near the intrusion, resulting in a higher rate of heat transfer from the intrusion to geothermal fluid, albeit with moderate fluid temperatures and enthalpies^4^,^5^,^6^,^16^. Intermediate permeability reduces the overall rate of heat transfer but leads to higher water temperatures and enthalpies^4^,^5^,^6^,^16^.

Heat transfer from a magmatic intrusion to geothermal water is controlled by a dramatic permeability decrease near the intrusion. Increasing temperature changes the mechanical behaviour of rock across the brittle–ductile transition (BDT)^17^,^18^,^19^, where the mode of deformation shifts from brittle fracturing to plastic flow, closing connected fluid flow pathways. The brittle–ductile transition temperature (*T*_BDT_) depends on the lithology, ranging from ∼360 °C for silicic rocks at typical crustal strain rates^5^,^18^,^20^ to as high as 800 °C for non-glassy basaltic rocks^21^,^22^. Below a permeability of ∼10^−16^ m^2^, heat transport changes from being advection- to conduction-dominated^4^,^5^,^6^, and any wells drilled into such conditions would encounter uneconomic rates of fluid production. Thus, the formation of potentially exploitable supercritical fluid resources hinges on whether hydrothermal fluid is heated to supercritical temperatures at conditions of sufficiently high rock permeability.

There currently exists no definition of what is considered a supercritical geothermal resource. Using practical and unambiguous criteria, we define potentially exploitable supercritical resources as those parts of a geothermal system where permeability is >10^−16^ m^2^ and temperature and specific enthalpy of water are greater than their critical values (373.976 °C and 2.086 MJ kg^−1^) (ref. 23). This definition avoids the distinction between ‘superheated' (fluid pressure below the critical pressure) and ‘supercritical' (above the critical pressure) resources, which was previously used in the analysis of the IDDP-1 well^12^,^13^ but is arbitrary as supercritical fluid properties vary gradually across the critical isobar. Rather, our definition allows analyzing the full continuum of supercritical resource conditions including the development of economically attractive resources at subcritical pressures such as IDDP-1.

We present the first numerical simulations of supercritical geothermal resource formation during the transient evolution of geothermal systems driven by magmatic intrusions. We demonstrate how primary geologic factors such as the brittle–ductile transition temperature, the host rock permeability and the intrusion depth control the extent and thermo-hydraulic structure of supercritical geothermal resources.

## Results

### Formation of supercritical geothermal resources

The formation of supercritical resources is a transient process in the evolution of geothermal systems (Supplementary Fig. 1; Supplementary Discussion). The results presented below refer to the phase during which the largest supercritical resources develop. It should be noted that during the evolution of the geothermal system the intrusion may have already crystallized when the hottest and most intense convection phases are encountered.

**Figure 1:**
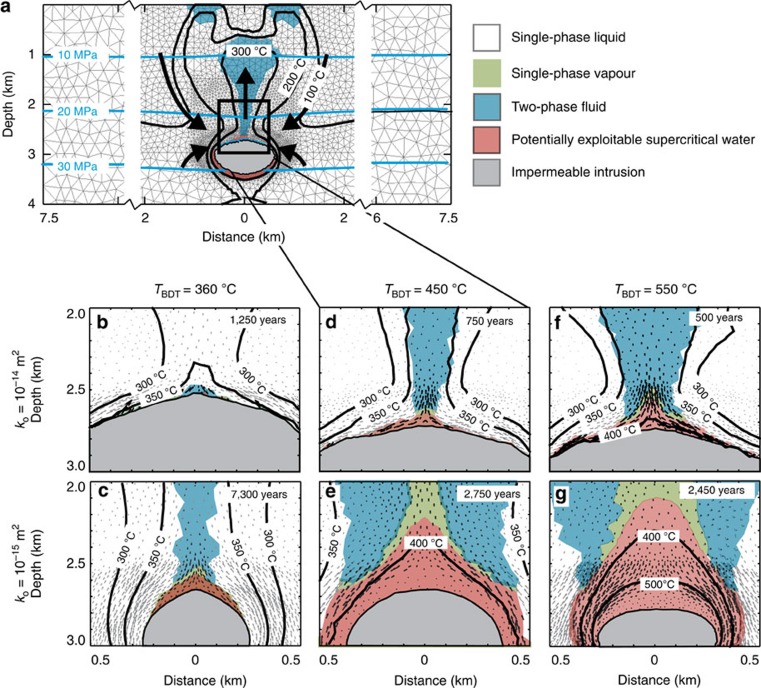
The formation of supercritical water resources depends on geologic controls. (**a**). Typical large-scale thermal structure of a simulated geothermal system, showing the fluid phase state distribution, temperature contours (black lines), fluid pressure contours (blue lines), potentially exploitable supercritical water resources (red areas, defined as fluid with temperature and specific enthalpy greater than 374 °C and 2.086 MJ kg^−1^, respectively, in host rock with a permeability >10^−16^ m^2^) as well as the impermeable intrusion (single-phase vapour at a permeability <10^−16^ m^2^, grey). Zones of two-phase (liquid and vapour) coexistence are shown in blue, and single-phase vapour at a temperature below the critical temperature is shown in green. The finite element grid, consisting of ∼10,000 triangular elements in a domain 5 and 15 km in horizontal and vertical extent, is also shown. (**b–g**). Snapshots of the area near the top of the intrusion (black box in **a**), under different conditions of host rock permeability (*k*_o_) and brittle–ductile transition temperature (*T*_BDT_). Liquid (grey) and vapour (black) flow vectors are also shown (not to scale between different fluid phases or simulations). We vary *k*_o_ from 10^−14^ m^2^ (**b**,**d**,**f**) to 10^−15^ m^2^ (**c**,**e**,**g**) and vary *T*_BDT_ from 360 °C (**b**,**c**), to 450 °C (**d**,**e**), and 550 °C (**f**,**g**).

The key control on the formation of supercritical resources is the brittle–ductile transition temperature *T*_BDT_. Extensive supercritical water resources can develop if *T*_BDT_ is at least 450 °C (Fig. 1d,e). Increasing *T*_BDT_ from 450 to 550 °C results in somewhat larger supercritical zones without dramatically changing the thermo-hydraulic conditions of such reservoirs (Fig. 1f,g). For *T*_BDT_<450 °C (for example, 360 °C, Fig. 1b,c) only minor supercritical resources develop because the threshold permeability is encountered at temperatures slightly higher than the critical temperature of water.

The extent and temperature of the supercritical resources strongly depend on host rock permeability. In high-permeability host rocks, the rate of convective water circulation surpasses the ability of the intrusion to heat most circulating water to supercritical temperatures, and supercritical water flow is confined to a thin (∼10 m) boundary layer on the perimeter of the intrusion that merges to form a focused upflow zone at the centre (Fig. 1d,f). Most of the supercritical resource only reaches temperatures of 375–400 °C, where the thermodynamic ability of supercritical water to transport heat is maximized^24^,^25^, even if *T*_BDT_ is as high as 550 °C. In contrast, supercritical resources in intermediate permeability systems are hotter (Fig. 1e,g). The water circulation rate near the intrusion is lower compared with high-permeability systems, so the conductive heat input across the brittle–ductile transition is sufficient to heat up a larger fraction of the circulating water to supercritical temperatures, and temperatures can approach *T*_BDT_ in the near vicinity of the intrusion. The supercritical resources can extend up to 700 m above the location of the *T*_BDT_ isotherm.

### Relation to conventional geothermal resources

**Figure 2:**
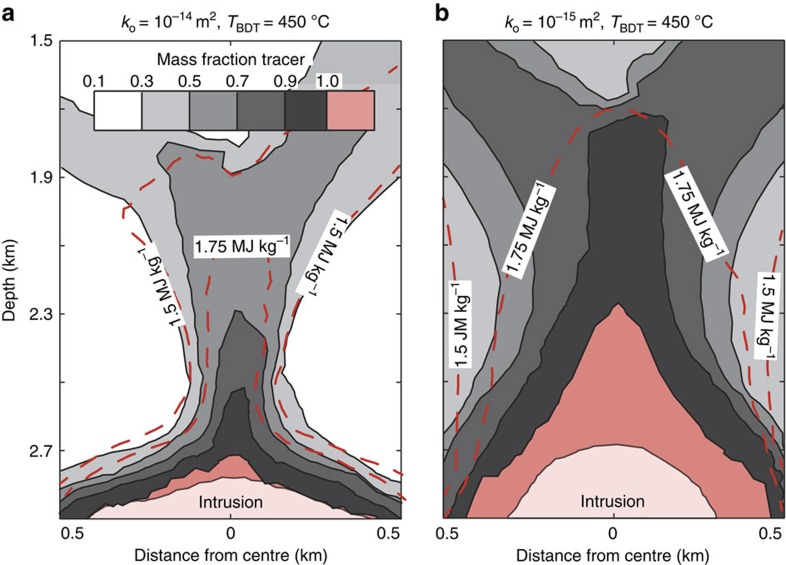
Supercritical reservoirs are related to conventional geothermal resources by fluid mixing. A passive tracer methodology was implemented to trace the flow fluid heated to supercritical conditions through the geothermal system. Colour scale indicates mixing (in terms of mass fraction) of fluid ascending out of the supercritical reservoir (mass fraction 1, red) and cooler liquid and/or vapour (grey tones). Results for the simulations shown in **a** (Fig. 1d) and **b** (Fig. 1e). Fluid specific enthalpy contours are shown with red dashed lines.

The results bring to light a close relationship between supercritical and conventional high-enthalpy geothermal resources. By tracing the flow of water from the supercritical resource (Methods), we find that conventional high-enthalpy geothermal resources form above supercritical resources as the ascending supercritical water progressively mixes with surrounding cooler water (Fig. 2). In high-permeability host rocks, the focused upflow of supercritical water above the centre of the intrusion mixes with significant amounts of cooler water, reducing water enthalpy to ∼1.5 MJ kg^−1^ at depths of 1.5–2 km (Fig. 2a). In intermediate permeability systems, a larger amount of ascending supercritical water is mixed with smaller amounts of cooler water, and accordingly the enthalpy of the geothermal system is greater (Fig. 2b). Host rock permeability therefore controls the enthalpy of geothermal systems by affecting both the size of the supercritical water reservoir and the mixing dynamics.

### Dependence on magma emplacement depth

The magma emplacement depth is an additional control on temperature and enthalpy in the supercritical resource and on the thermal structure in the overlying geothermal system, since supercritical water properties vary with pressure. The simulations in Fig. 1 featured an intrusion that was initially emplaced with an upper depth at 2.5 km. Supplementary Fig. 2 gives an example of how model results change if the emplacement depth of the intrusion is shifted 0.5 km up or down. If the depth to the top of the intrusion is at 2 km depth, the fluid pressure above the intrusion is less than the critical pressure of water (22.055 MPa)^23^. In contrast, in systems in which the intrusion is situated at 3 km depth, the fluid pressure above the intrusion is greater than the critical pressure. At 400 °C, the specific enthalpy of water decreases from ∼3 MJ kg^−1^ at 15 MPa (corresponding to hot hydrostatic pressure at a depth near 2 km) to ∼2.5 MJ kg^−1^ at 25 MPa (3 km), affecting the capacity of water to advect heat on ascent. However, changing magma emplacement depth can result in different convection cell geometries (Supplementary Discussion), which precludes drawing universally applicable conclusions.

## Discussion

**Figure 3:**
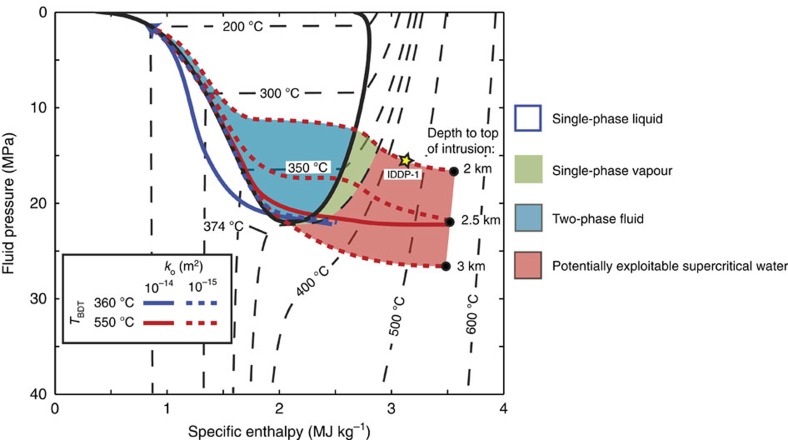
The thermal structure of high-enthalpy geothermal systems. Pressure–enthalpy ascent paths were extracted from selected simulations shown in Fig. 1 and Supplementary Fig. 2, and are superimposed onto a phase diagram of water showing the region of two-phase liquid and vapour coexistence and isotherms. The areas of potentially exploitable supercritical fluid, single-phase vapour and two-phase fluid are shown in red, green, and blue, respectively. The measured reservoir temperature and enthalpy for the IDDP-1 well (450 °C, 3.2 MJ kg^−1^)^11^ is shown with a yellow star.

The combined effects of *T*_BDT_, host rock permeability and intrusion depth on supercritical resources and on the thermal structure of geothermal systems can be summarized in a pressure–enthalpy (*p*–*h*) diagram (Fig. 3). In systems with a low *T*_BDT_ and high host rock permeability (blue solid line), enthalpy is reduced to ≤1.5 MJ kg^−1^ after mixing, giving rise to a geothermal system with boiling restricted to shallow depths. In systems with a high *T*_BDT_ (red lines) and/or intermediate permeability (dotted lines), the higher enthalpy input and lower degree of mixing leads to geothermal systems that boil over the entire depth range above the supercritical resource. The transition from supercritical to boiling conditions occurs over a small pressure range in high-permeability systems and more gradually in intermediate permeability systems, reflecting the mixing dynamics.

Our models explain the conditions encountered in the IDDP-1 well as the natural result of drilling into a shallow magmatic intrusion hosted in basaltic rock, even though the 900 °C hot magma itself was rhyolitic^15^. The measured reservoir conditions^13^ (Fig. 3, yellow star) lie directly on the *p*–*h* ascent path of a system with a 2-km deep intrusion in rocks with *T*_BDT_ of 550 °C and an intermediate permeability of 10^−15^ m^2^, values which are appropriate for the Krafla system^21^,^26^. This correspondence corroborates our model results as well as the conclusion that supercritical geothermal resource properties depend on the primary geologic controls. The *p*–*h* relations further suggest that IDDP-1 drilled into a supercritical resource at close to the optimum conditions expected in the range of geologic parameters considered. Water at a similar temperature at greater depth would have a lower enthalpy, which might lead to unwanted liquid condensation upon near-isenthalpic depressurization during production from a wellbore but can be avoided if the specific enthalpy of the supercritical resource is greater than ∼2.8 MJ kg^−1^ (Fig. 3).

The *p*–*h* ascent paths (Fig. 3) converge on a trajectory corresponding to ascending boiling liquid with a vapour fraction ranging from 0 to 5% by mass, consistent with observations from high-enthalpy geothermal systems^27^,^28^. Knowledge of water enthalpy distribution in a boiling geothermal system may guide future exploration for supercritical resources, since it varies with changing intrusion depth, brittle–ductile transition temperature and host rock permeability (Fig. 3). However, systems with saline geothermal water may behave differently, as the addition of salt greatly extends the temperature–pressure conditions of boiling^29^, potentially shifting the location of comparable resources to greater depth (for example, to depths equivalent to >30 MPa hot hydrostatic head for water with seawater salinity such as in the Reykjanes system in Iceland).

Geothermal energy will become more important as the world transitions to a less carbon-intensive energy infrastructure. By exploiting supercritical water resources, the energy yield per well is up to an order of magnitude higher^10^,^11^,^12^, massively improving the economics of geothermal electricity production. Our results also provide a missing conceptual understanding^30^ of how geologic factors control the abundance and thermo-hydraulic state of high-enthalpy and supercritical water resources, so that future attempts to discover and exploit supercritical water resources may be more successful. Although the effect of other factors such as water salinity^31^, mineral precipitation/dissolution^32^, magmatic fluid production^33^ and stress state-dependent permeability^33^ remain to be investigated, our models demonstrate that sizeable supercritical resources may form naturally in many magma-driven geothermal systems. The efficiency of heat transport by convecting groundwater is optimized at near-critical temperatures^24^,^25^ and, taken together, this more generally implies that supercritical water plays a key role in removing heat from magmatic intrusions. The strain rate dependence of *T*_BDT_ suggests that this may not be restricted to basaltic systems but may pertain also to silicic rocks if tectonic deformation rates are high enough^20^. While other challenges to successful power production from supercritical resources still need to be addressed, such as how to chemically treat the corrosive and silica-rich supercritical fluid^34^, our models suggest that geologic conditions in most high-enthalpy geothermal fields allow the formation of potentially lucrative target resources.

## Methods

### Model set-up

We use the CSMP++ platform^35^ to model fluid flow and heat transfer in a generic two-dimensional geometry featuring an initially 900 °C hot intrusion in a hydrostatically pressured porous medium. The intrusion initially has an elliptical geometry with major and minor axes lengths of 2 and 1 km and is modelled as an instantaneously emplaced hot body within a background conductive temperature gradient. Repeated intrusion and/or the effect of replenishment have not been included in the simulations. The left, right and bottom boundaries are no-flow, while the top boundary is treated as open, allowing fluids to discharge or recharge as needed in order to maintain a constant pressure of 1 bar. The temperature at the top boundary is allowed to vary based on the enthalpy of upflowing fluids, or in the case of recharge, the recharging liquid has a fixed temperature of 10 °C. The fluid is assumed to be pure water according to the equation of state of ref. 23. Initial rock and fluid properties are shown in Supplementary Tables 1 and 2, respectively. The permeability of the host rock is homogenous and isotropic, and is varied between simulations from 10^−14^ to 10^−15^ m^2^. To mimic the effect of the brittle–ductile transition, we adopt a formulation^5^ of temperature-dependent permeability in which permeability decreases log-linearly with increasing temperature above a specified BDT onset temperature, *T*_BDT_, which depends on the rock type. In this model, the 10^−16^ m^2^ threshold is reached ∼25–30 °C above *T*_BDT_. We investigate the effect of *T*_BDT_ being at 360 (representing silicic rocks at typical crustal strain rates^5^,^18^,^20^), 450 and 550 °C (non-glassy basaltic rocks^21^,^22^).

### Computational method and governing equations

The governing equations of multiphase mass and energy conservation are solved using a continuum porous medium approach with a pressure–enthalpy-based formulation for energy transport with a control volume finite element method (CVFEM)^35^. Conservation of fluid mass is given by:


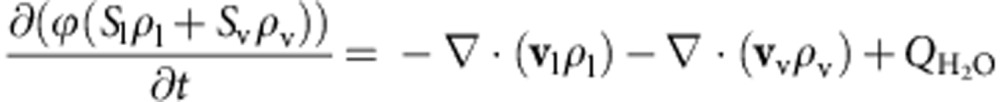


where *ϕ* refers to the porosity, *S*, *ρ* and **v** refer to the volumetric saturation, density and Darcy velocity, respectively, of liquid l or vapour v, and 
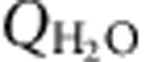
 denotes a source term. Phase velocities were obtained using an extended two-phase form of Darcy's law:


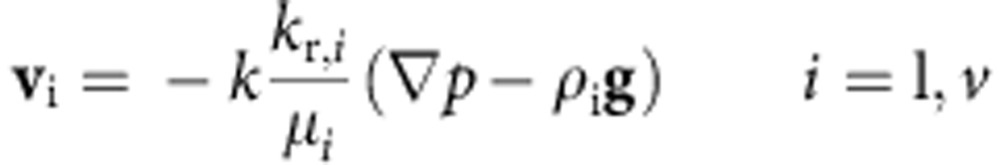


*k* denotes permeability, *k*_r_ relative permeability of fluid phase *i*, *μ* dynamic viscosity*, p* total fluid pressure, and **g** gravitational acceleration vector. A linear relative permeability model with a liquid residual saturation of 0.3 and vapour residual saturation of zero is adopted^5^.

Conservation of energy accounts for conduction of heat in the rock and advection of enthalpy by the fluid:


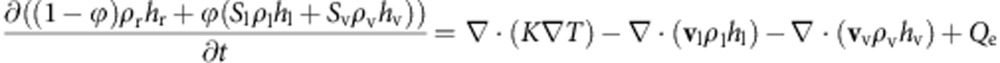


where the subscript r refers to the rock, *h*_*i*_ denotes the specific enthalpy of the phase indicated, *K* is thermal conductivity, *T* is temperature and *Q*_e_ is a source term. Thermal equilibrium between fluid and rock at a given node in the mesh and at each time step is ensured by iterating temperature at a constant pressure and redistributing the total enthalpy between rock and fluid according to their thermodynamic properties until they each have the same temperature.

We implemented a passive tracer method to follow water heated to supercritical conditions through the geothermal system (Fig. 2). The mass fraction of tracer is set to one within a package of water at supercritical conditions (*T*>374 °C, *h*>2.086 MJ kg^−1^), and becomes proportionally smaller when this water mixes with cooler water (tracer concentration zero) upon ascent.

## Additional information

**How to cite this article**: Scott, S. *et al*. Geologic controls on supercritical geothermal resources above magmatic intrusions. *Nat. Commun*. 6:7837 doi: 10.1038/ncomms8837 (2015).

## Supplementary Material

Supplementary Information Supplementary Figures 1-2, Supplementary Tables 1-2, Supplementary Discussion and Supplementary Reference
