# Supplementary material for: Geologic controls on supercritical geothermal resources above magmatic intrusions
Source: Nat Commun. 2015 Jul 27;6:7837. doi: 10.1038/ncomms8837 (PMC4525172; doi:10.1038/ncomms8837)
Supplement: Supplementary Information — Supplementary Figures 1-2, Supplementary Tables 1-2, Supplementary Discussion and Supplementary Reference [file ncomms8837-s1.pdf]

Supplementary Figures

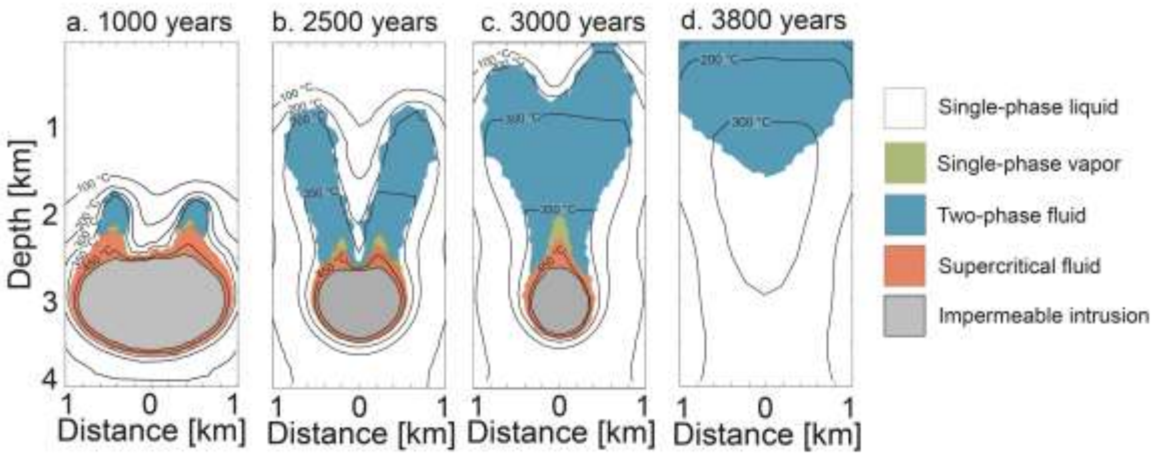

Supplementary Figure 1. Transient behavior of geothermal systems. The style of convection in our simulations shows strong transient effects as the intrusion progressively cools and the two upflow zones initially developing on the margins of the intrusion merge together. **a**.-**d**. A simulation with a host rock permeability $k_0 = 10^{-15}$ m^2 and a brittle-ductile transition temperature T_{BDT} = 450 °C (shown in Figure 1e, text) is compared at various times (**a**. 1000 years, **b**. 2500 years, **c**. 3000 years, **d**. 3800 years).

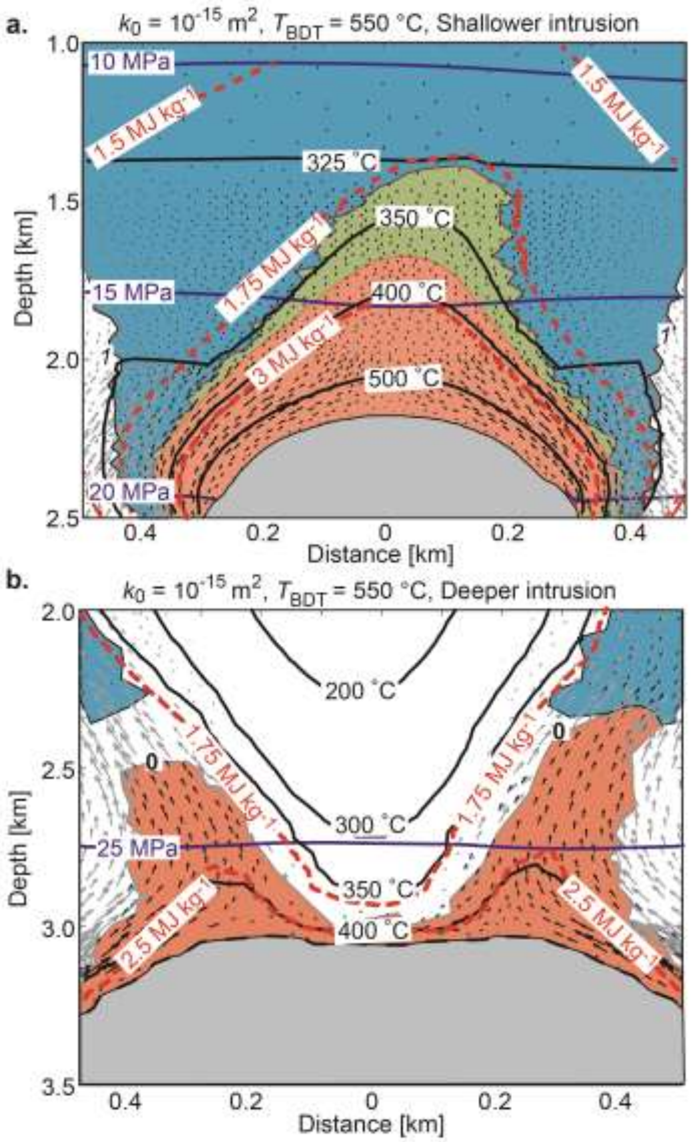

Supplementary Figure 2. Complex role of magma emplacement depth. The depth of magma chamber emplacement influences the number and geometrical configuration of upflow zones in geothermal systems. This figure compares an intrusion emplaced with a top at 2 (**a**) or 3 (**b**) km depth, with an host rock permeability $k_0 = 10^{-15}$ m^2 and a brittle-ductile transition temperature T_{BDT} = 550 °C. Fluid pressure (blue lines), temperature (black lines), and fluid specific enthalpy (red dashed lines) are shown. Two-phase zones, liquid and vapor flow vectors and areas of potentially exploitable supercritical fluid and the impermeable intrusion are also shown according to the coloring scheme shown in Supplementary Figure 1.

Supplementary Tables

Supplementary Table 1. Initial rock properties.

Initial rock property	Symbol	Host rock	Magma chamber	Unit
Temperature	T	~45°C/km depth	900	°C
Porosity	ϕ	0.1	0.05	-
Permeability	k	Temperature-dependent: see text		m^2
Heat capacity (isobaric)	$c_{p,\mathrm{r}}$	880	2,000	J kg^{-1} $°C^{-1}$
Compressibility	β_r	10^{-20}	10^{-20}	Pa^{-1}
Density	ρ_r	2,750	2,750	kg m^{-3}
Thermal conductivity	K	2.25	2.25	W m^{-1} $°C^{-1}$

Supplementary Table 2. Initial fluid properties.

Initial fluid property	Symbol	Value	Unit
Temperature	T	+45°C/km depth	°C
Pressure	P	Hydrostatic<br>(in magma chamber: lithostatic)	Pa
Compressibility	β_i	Equation of state (EOS)[1]	Pa^{-1}
Density	ρ_i	EOS[1]	$kg\ m^{-3}$
Dynamic viscosity	μ_i	EOS[1]	Pa s
Specific enthalpy	h_i	EOS[1]	$J\ kg^{-1}$

Supplementary Discussion

Transient effects on supercritical geothermal resource development

The snapshots shown in Figure 1 (text) represent a stage in the transient evolution of a simulated geothermal system. In order to allow the model results between different simulations to be compared directly, the time was chosen so that the system features a single upflow zone centred over the intrusion, rather than multiple upflow zones located on the margins of the intrusion. Supercritical resources extend to a somewhat shallower depth if a single central upflow zone is present. This is illustrated in Supplementary Figure 1. For the example shown in Supplementary Figure 1, we chose the time to analyse model results for presentation in Figure 1 (text) to be between **b**. and **c**., right after the two upflow zones have merged and the supercritical resource is largest. We adopted a similar practice for the other simulations.

Effect of magma emplacement depth

Throughout most of the text, we discuss results for an intrusion whose top is at a depth of 2.5 km. However, varying magma emplacement depth within a relatively small range has strongly effects on the thermal structure of supercritical resources and geothermal systems due to the pressure-dependence of water properties. Simulation with a depth to the top of the intrusion of 2 km and 3 km are compared in Supplementary Figure 2. In systems in which the depth to the top of the intrusion is at 2 km depth, the fluid pressure above the intrusion is less than the critical pressure of water (22 MPa)[1], ascending supercritical fluid cools and depressurizes to form a vapor-rich two-phase zone (Supplementary Figure 2a). In contrast, in systems in which the intrusion is situated at 3 km depth, the fluid pressure above the intrusion is greater than the critical pressure, and ascending supercritical fluid contracts to a liquid or a liquid-rich two-phase zone with a lower fluid enthalpy (Supplementary Figure 2b). Additionally, Supplementary Figure 3 shows that an intrusion with a depth a 2 km develops a single upflow zone centered over the intrusion, while an intrusion with a depth of 3 km develops two upflow zones on the margins of the intrusion that do not merge until relatively late in the evolution of the system. A full discussion of the effect of intrusion depth merits closer attention, and is beyond the scope of this study.

Supplementary References

1. Haar, L. Gallagher, J. S. & Kell, G. *NBS/NRC Steam Tables*. Hemisphere, Washington, D. C. (1984)
